# The frequency of psychotic symptoms in types of dementia: a systematic review

**DOI:** 10.1590/1980-5764-DN-2022-0044

**Published:** 2023-04-14

**Authors:** Rebeca Mendes de Paula Pessoa, Madson Alan Maximiano-Barreto, Letícia Lambert, Érica Dayanne Meireles Leite, Marcos Hortes Nisihara Chagas

**Affiliations:** 1 Universidade de São Paulo, Departamento de Neurociências e do Comportamento, Ribeirão Preto SP, Brazil.; 2 Universidade Federal de São Carlos, Grupo de Estudos e Pesquisas em Saúde Mental, Cognição e Envelhecimento, São Carlos SP, Brazil.; 3 Instituto Bairral de Psiquiatria, Itapira SP, Brazil.

**Keywords:** Hallucinations, Delusions, Dementia, Prevalence, Geriatric Psychiatry, Systematic Review, Alucinações, Delusões, Demência, Prevalência, Psiquiatria Geriátrica, Revisão Sistemática

## Abstract

The frequency of psychotic symptoms in older adults is high, mainly in neurocognitive cognitions of the most varied etiologies.

**Objectives:**

This study aimed to review the studies that analyze the frequency of the types of delusions, hallucinations, and misidentifications in dementia conditions of different etiologies.

**Methods:**

A systematic review was conducted on August 9, 2021, in the PubMed, PsycInfo, Embase, Web of Science, and Scopus databases with the following descriptors: (dementia OR alzheimer disease OR dementia with Lewy bodies OR frontotemporal dementia OR mixed dementia OR vascular dementia OR major neurocognitive disorder OR parkinson disease dementia) AND (psychotic symptoms OR psychosis OR hallucinations OR delusions OR psychopathology OR misidentification) AND (prevalence OR epidemiology).

**Results:**

A total of 5,077 articles were found, with a final inclusion of 35. The overall frequency of psychotic symptoms ranged from 34 to 63% in dementia conditions of the most varied etiologies. Alzheimer’s disease (AD) presents more delusions and hallucinations and has a higher frequency regarding the presence of misidentifications. On the contrary, Dementia with Lewy bodies (DLB) seems to present more hallucinations, even auditory, when compared to the other dementias, concomitantly with delusions. Vascular and frontotemporal dementia present fewer psychotic symptoms than DLB and AD.

**Conclusions:**

We identified a gap in the literature on the description of the psychotic symptoms of dementia, mainly in those of non-AD etiologies. Studies that assess the neuropsychiatric symptoms of dementias deeply might contribute in a more definite manner to the causal diagnosis of dementia.

## RESUMO

A frequência de sintomas psicóticos em idosos é alta, principalmente em cognições neurocognitivas das mais variadas etiologias.

**Objetivos:**

Revisar os estudos que analisam a frequência dos tipos de delírios, alucinações e erros de identificação em quadros demenciais de diferentes etiologias.

**Métodos:**

Foi realizada uma revisão sistemática em 9 de agosto de 2021, nas bases de dados PubMed, PsycInfo, Embase, Web of Science e Scopus, com os seguintes descritores: (demência OR doença de alzheimer OR demência com corpos de Lewy OR demência frontotemporal OR demência mista OR vascular demência OU transtorno neurocognitivo maior OU demência da doença de Parkinson) E (sintomas psicóticos OU psicose OU alucinações OU delírios OU psicopatologia OU identificação errônea) E (prevalência OU epidemiologia).

**Resultados:**

Foram encontrados 5.077 artigos, com inclusão final de 35. A frequência geral de sintomas psicóticos foi de 34 a 63% em quadros demenciais das mais variadas etiologias. A doença de Alzheimer (DA) apresenta mais delírios, alucinações e maior frequência quanto à presença de erros de identificação. Por outro lado, a demência com corpos de Lewy (DCL) parece apresentar mais alucinações, inclusive auditivas, quando comparada às demais demências, concomitantemente aos delírios. As demências vascular e frontotemporal apresentam menos sintomas psicóticos do que a DCL e a DA.

**Conclusões:**

Identificamos lacuna na literatura quanto à descrição dos sintomas psicóticos das demências, principalmente naquelas de etiologia não DA. Estudos que aprofundem os sintomas neuropsiquiátricos das demências podem contribuir de forma mais definitiva para o diagnóstico causal da demência.

## INTRODUCTION

The frequency of psychotic symptoms seems to increase with the aging process. An 11% increase in the annual incidence of these symptoms is estimated for every 5-year increase in age^1^. The high prevalence of these symptoms is noteworthy, especially in patients with neurocognitive disorders; this group is called behavioral and psychological symptoms of dementia (BPSD). BPSD is a group of heterogeneous manifestations that arise in the course of dementia and are related to changes in perception, thought content, mood, or behavior^2,3^. Due to the increased prevalence of Alzheimer’s disease (AD), the International Psychogeriatric Association (IPA) revised in 2021 the criteria for psychosis in neurocognitive disorders. For the diagnosis, consideration should be given to the presence, lasting at least 1 month, of delusions or hallucinations (visual or auditory); the diagnosis of a neurocognitive disorder that presents a chronological relationship with these symptoms; and the loss in functionality. The presence of other primary psychiatric disorders, delirium, and other medical conditions and psychotic symptoms within a cultural context should be excluded^4^.

Psychotic symptoms can present different etiologies. Despite that, most of the studies tend to group them, generating less clarity on the effects of these symptoms^5^. In addition, the associations between the types of psychotic symptoms and the etiology of the base conditions, in particular those of a neurodegenerative etiology, are not well-documented in the literature^6^.

In relation to the main psychotic symptoms, we can cite delusions and hallucinations^7^. Delusions are false or incorrect beliefs about reality, firmly held despite evidence to the contrary, and can be classified according to their content (e.g., persecutory, reference, and somatic delusions, delusions of grandeur and others). Hallucinations are changes in the sense perception that seem real, but that are not caused by external stimuli relevant to the sensorium, and can occur in any sensory modality^8^. Misidentifications (MIs) are behavioral and psychological symptoms also found in dementia^9^ and include any change in the recognition or interpretation of events, people, or things^10^. Examples of MIs are Capgras (imposter) syndrome, phantom boarder (someone uninvited in his home), and reduplication of people and places^11^.

The prevalence of psychotic symptoms varies according to the etiology of dementia^2,3,12^. However, the main types of psychotic symptoms, especially in an older population, are still a challenge, and it is not clear whether psychopathological differences are more prevalent in a particular etiology of dementia^13^. A difference in the psychotic symptoms could assist in this differential diagnosis^13^.

Considering that there is a gap in the literature of studies that assess psychotic symptoms in detail in the various etiologies of dementia, this article aimed at systematically reviewing the studies that analyzed the frequency of the types of delusions, hallucinations, and MI in dementia cases of different etiologies.

## METHODS

A systematic review of the national and international literature, regardless of publication date, about the types of delusions, hallucinations, and MIs in dementia conditions was carried out on August 9, 2021. To such end, a search was conducted with the following keywords: (dementia OR alzheimer disease OR dementia with Lewy bodies OR frontotemporal dementia OR mixed dementia OR vascular dementia OR major neurocognitive disorder OR parkinson disease dementia) AND (psychotic symptoms OR psychosis OR hallucinations OR delusions OR psychopathology OR misidentification) AND (prevalence OR epidemiology). Studies indexed in the following databases were researched: PubMed, PsycInfo, Embase, Web of Science, and Scopus. This systematic review was submitted to PROSPERO: CRD42020205752.

The inclusion criteria were studies with a sample including dementia with delusions, hallucinations, or MIs and those describing the types and frequency of these symptoms in the results. The revised IPA criteria for psychosis in neurocognitive disorders were considered^4^. The articles were included, regardless of their language. The exclusion criteria were studies with dementia of infectious etiologies, case reports, letters to the editor, book chapters and reviews, collections of abstracts, comments, notes, errata, theses or dissertations, or bibliographic/systematic reviews; case reports; studies with cognitive declines with a psychiatric etiology; studies with mild cognitive impairment; and studies without any etiological description of dementia. No time limit was adopted.

Independently, the authors searched and extracted the following data from the selected articles: author, year of publication, type of dementia and criteria used for diagnosis, characteristics of the sample (gender, mean age, schooling, severity of dementia), instrument used for the assessment of the symptoms, frequency of delusions, MIs and hallucinations, detailed description of the psychotic symptoms, and other correlations found in the studies.

The Strengthening the Reporting of Observational Studies in Epidemiology (STROBE) instrument was used for the methodological assessment of the studies, as they were of the observational type^14^. STROBE is an instrument that assesses the reporting quality of the study with a maximum score of 22, with no cutoff point. Scoring of the items is directly proportional to the methodological quality of the study. A percentage of the instrument’s items contemplated for each study was calculated. The methodological analysis was not used as a criterion to exclude articles. The *Rayyan* website was used in the selection of the articles. The data were reviewed, and any and all disagreements were discussed among the authors.

## RESULTS

### Characteristics of the studies

**Figure 1.**
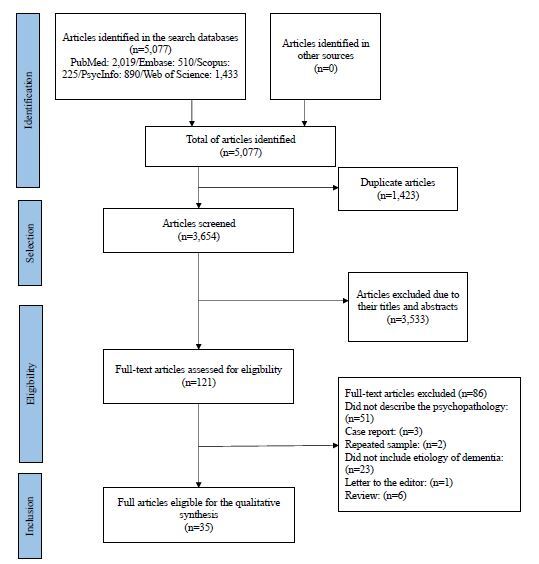
PRISMA flowchart.^15^

A total of 5,077 articles were identified, of which 3,654 remained after exclusion of duplicates. The titles and abstracts of these articles were evaluated, with the exclusion of 3,533. Of note, 124 articles were read in full, with the final selection of 35 according to the inclusion and exclusion criteria. Figure 1 illustrates the process carried out according to the recommendations of the PRISMA (Preferred Reporting Items for Systematic Reviews and Meta-Analyses) group^15^.

All the studies were observational, 21 were cross-sectional, 6 were prospective, 7 were retrospective, and 1 was cross-sectional and prospective. Regarding the type of dementia under study, 30 articles assessed AD, 5 addressed vascular dementia (VD), 9 assessed dementia with Lewy bodies (DLB), and 5 evaluated frontotemporal dementia (FTD). Ten articles addressed several causes of dementia altogether.

In relation to the methodological analysis performed with the STROBE instrument, all the articles met more than 70% of the instrument’s items. Four (12.5%) articles covered from 70 to 80% of the items, 23 (62.5%) articles included from 80 to 90% of the items, and 8 (25%) articles contemplated more than 90% of the items.

The main reasons for not covering the items were the absence of the description of the type of study in the title and abstract (item 1) in 27 (77%) articles, omission of the data collection date (item 5) in 18 (51%) articles, incomplete description of the statistical methods (item 12) in 29 (83%) articles, absence of sample calculation (item 10) in all (100%) the articles, absence of a flow diagram about selection of the participants (item 13) in 33 (94%) articles, and no specification of the study funding (item 22) in 13 (37%) articles.

### Alzheimer’s disease

A total of 30 articles dealing with AD were included (Table 1)^5–7,9,11,16–45^. The most commonly used diagnostic method was the criteria of the National Institute of Neurological and Communicative Disorders and Stroke-Alzheimer’s Disease and Related Disorders Association (NINCDS-ADRDA) alone, in 14 (48%) articles. The other articles used the following diagnostic methods: *Diagnostic and Statistical Manual of Mental Disorders* (*DSM*) and NINCDS-ADRDA (17%; n=5), clinical evaluation (14%; n=4), neuropathological (7%; n=2), only *DSM* (3.5%; n=1), National Institute on Aging-Alzheimer’s Association (NIA-AA) (3.5%; n=1), autopsy (3.5%; n=1), and unspecified (3.5%; n=1).

**Table 1.** Description of the psychotic symptoms in the articles selected.

Author	Etiology of dementia	Sample (n)	Frequency of psychosis (% of total sample)	Description of the symptoms (% of total sample)
Binetti et al.^16^	AD/VD	92 AD: 61 VD: 31	Delusions: AD: 46%; VD: 39% Misidentifications: AD: 37.7%; VD: 19%	Delusions: AD – Persecutory: 13%; Theft: 13%; Jealousy: 6% VD – Persecutory: 23%; Theft: 23%; Jealousy: 3%; Somatic: 3%
van de Beek et al.^17^	DLB	73	Delusion: 9.5 Hallucinations: 11%	Delusions: Paranoid: 2.7%; Capgras: 5.5%; Somatic: 1.4%; Guilt: 1.4% Hallucinations: Tactile: 4.1%; Olfactory: 7.3%; Auditory: 11%
Chiu and Chung^18^	AD	91	Delusions: 35.2%	Delusions: Theft: 27.5%; Persecutory: 15.4%; Not being home: 5.5%; Abandonment: 4.4%; Jealousy: 2.2%; Intruder: 2.2%; Media people in the house: 2.2%; Capgras: 1.1%
Cohen-Mansfield et al.^19^	AD	74	Delusions: 46%	Delusions: Theft: 22%; Persecutory: 9%/Not being home: 7%; Abandonment: 5%; Non-paranoid: 5%
Cummings et al.^20^	AD/VD	45 AD: 30 VD: 15	Psychosis: AD: 46%; VD: 46% Hallucinations: AD: 3.3%; VD: 27%	Hallucinations: AD – Olfactory: 3.33% VD – Auditory: 13%; Visual: 20% Misidentifications: AD – Capgras: 7%; Phantom Boarder: 3.33% VD – Phantom Boarder: 7%
Deutsch et al.^7^	AD	170	Delusions: 43.5% Hallucinations: 23.5% Misidentifications: 30%	Delusions: Persecutory: 32%; Reference: 6%; Jealousy: 4%; Grandeur: 0.6; Somatic: 0.6% Hallucinations: Visual: 20%; Olfactory: 11%; Tactile: 0.6% Misidentifications: Not being home: 15%; Phantom Boarder: 9%; Mirror: 6%; Capgras (caregiver): 0.6%
Devenney et al.^21^	FTD-BV FTD-ALS	Total: 79 Dementia: 56 FTD-BV: 36 FTD-ALS: 20	Psychosis: 34% Delusions: 28.57% Hallucinations: 25%	Delusions: Persecutory: 18%; Somatic: 9%; Grandeur: 7%; Jealousy: 5% Hallucinations: Auditory: 9%; Visual: 9%; Tactile: 4%
Farber et al.^22^	AD	109	Psychosis: 63% Delusions: 60% Hallucinations: 32% (isolated: 4%) Misidentifications: 31% (84% with another psychosis)	Delusions: Persecutory: 37%; Jealousy: 3%; Others: 33% Hallucinations: Visual: 25%; Olfactory: 13%; Others: 6% Misidentifications: Other people: 23%; Person himself/herself: 5%; Television: 2%
Flynn et al.^23^	AD/VD	33 AD: 19 VD: 14	Delusions: Total: 60.6% AD: 68.4% VD: 50%	Delusions: AD – Theft: 58%; Not being home: 47%; Persecutory: 37%; Capgras: 37% Abandonment: 37%; Jealousy: 26%; Phantom Boarder: 21% VD – Theft: 21%; Not being home: 14%; Jealousy: 14%; Phantom Boarder: 14%; Capgras: 7%; Abandonment: 7%
Förstl et al.^24^	AD	56	Delusions: 16% Misidentifications: 25%	Delusions: Persecutory: 7%; Jealousy: 4%; Theft: 2%; Poisoning: 2%; Nihilist: 2% Hallucinations: Auditory: 12.5%; Visual: 17.8% Misidentifications: Capgras: 16%; Phantom Boarder: 9%; Television: 4%; Mirror: 4%
Geroldi et al.^25^	AD	41	Delusions: 46%	Delusions: Theft: 46%; Persecutory or jealousy: 15%
Gormley and Rizwan^26^	AD	70	Psychosis: 36% Delusions: 34% Hallucinations: 11%	Delusions: Persecutory: 21%; Theft: 14%; Jealousy: 11%; Abandonment: 7%; Capgras (caregiver): 4%; Not being home: 6%; Others: 6% Hallucinations: Visual: 7%; Auditory: 4%
Hamuro et al.^27^	AD	202	Delusions: 29.7% Hallucinations: 8.42% Misidentifications: 21.8%	Delusions: Insult: 26%; Theft: 21%; Jealousy: 2%; Poisoning: 1.4%; Persecutory: 0.5%; Observation: 0.5%; Others: 3% Hallucinations: Visual: 7.43%; Auditory: 2.97% Misidentifications: Phantom Boarder: 20%; Capgras: 1.4%; Mirror: 0.5%
Harciarek and Kertesz^28^	AD, FTD-BV, PPA, SD, CBD, PSP, PD, DLB	PPA: 60 SD: 17 PSP: 13 DLB: 36 PD: 9	Misidentifications: AD: 15.8% DLB: 16.3% SD: 8.3%	AD – Capgras: 5.9%; Capgras + Phantom Boarder or environment reduplication: 7.4%; Phantom Boarder or environment reduplication: 1.5% DLB – Capgras alone: 8.3%; Capgras + Phantom Boarder: 8.3% SD – Capgras: 2%; Capgras + Phantom Boarder/reduplication of place: 2% No relationship between MIs and FTD-BV, PPA, PSP, PD.
Ikeda et al.^29^	AD	112	Delusions: 47.3% Misidentifications: 23%	Delusions: Theft: 75.5%; Persecutory: 9%; Abandonment: 2%; Jealousy: 5%; Others: 9% Misidentifications: Phantom Boarder: 14%; Not being home: 4%; Television: 4%/Capgras: 1%
Korhonen et al.^30^	AD/FTD-BV	83 AD 75 FTD-BV	Delusions: AD: 4.8% FTD-BV: 34.67% Hallucinations: AD: 4.8% FTD-BV: 16%	AD – Auditory: 1.2%; Visual: 3.6% FTD – Auditory: 8%; Visual: 13.3%
Kwak et al.^31^	AD	230	Delusions: 27.4% Misidentifications: 5.2%	Delusions: Theft: 15%; Jealousy: 5%; Persecutory: 4%; Abandonment: 4%; Erotic: 0.4%; Grandeur: 0.4%; Mixed: 5% Misidentifications: Capgras: 4%; Phantom Boarder: 3%; Reduplication of place: 3%; Television: 2%; Mirror: 1%
Leroi et al.^32^	AD/VD	260	Psychosis: AD: 26.9%/VD: 15.3% Delusions: AD: 21.8%/VD: 12.5% Hallucinations: AD: 12.8%/VD: 15.5% Misidentifications: AD: 8%/VD: 6.8%	Delusions: AD – Persecutory: 6.6%; Theft: 12.7%; Jealousy: 2.2%; Abandonment: 4.1%; Phantom Boarder: 4.6%; Capgras: 1.6%; Not being home: 3.1%; Media people in the house: 2% VD – Theft: 6.8%; Abandonment: 3.4%; Phantom Boarder: 5.1%; Capgras: 5.1%; Not being home: 3.4%; Media people in the house: 1.7% Hallucinations: AD – Auditory: 3.6%; Visual: 9.1%; Tactile: 1%; Gustatory: 0.5% VD – Auditory: 6.9%; Visual: 8.5%
Linszen et al.^33^	AD	1,227	Delusions: 2% Hallucinations: 4.5%	Delusions: Theft: 22%; Capgras: 0.8%; Persecutory: 0.7% Hallucinations: Visual: 3%; Auditory: 1%; Olfactory: 0.4%; Tactile: 0.2%; Not specified: 1%
Liu et al.^34^	AD	142	Delusions: 50.7% Hallucinations: 24.6%	Delusions: Theft: 30.3%; Persecutory: 29.6%; Not being home: 14.7%; Capgras (caregiver): 3.5%; Abandonment: 4.2%; Jealousy: 4.2%; Others: 1.4% Hallucinations: Visual: 21.1%; Auditory: 12%; Olfactory: 0.7%; Tactile: 0.7%
Mendez et al.^35,36^	AD	217	Delusions: 30% Hallucinations: 25.4% Misidentifications: 25.4%	Delusions: Theft: 11%; The other is dead: 5.5%; Persecutory: 5.5%; Jealousy: 2.5%; Somatic: 2%; Intrusion: 1.3%; Duplicate residence: 0.5%; Abandonment: 0.5% Hallucinations: Visual: 19%; Auditory: 3%; Tactile: 1%; Auditory+Visual: 3% Misidentifications: Capgras: 5%; Phantom Boarder: 5%; Mirror: 2.3%; Prosopagnosia: 1.4%; Television: 0.5%; Double subjective autoscopy: 0.5%
Mendez et al.^6^	AD/FTD	AD: 23 FTD: 86	Delusions AD: 17.4%/FTD: 2.3% Hallucinations AD: 21.7%/FTD: 3.48%	Delusions: AD – Delusions of jealousy: 17.4% FTD – Delusions of having been married to famous people and that her husband treated her badly; 1% with paranoid delusion
Migliorelli et al.^37^	AD	103	Delusional disorder: 20% Hallucinations: 3%	Delusions: Theft: 14%; Hypochondriac: 14%; Abandonment: 7%; Not being home: 6%; Capgras: 6%; Grandeur: 6%; Jealousy: 4%; Erotomania: 2%
Mizrahi et al.^38^	AD	771	Delusions: 32% Hallucinations: 7%	Delusions: Theft: 22%; Grandeur: 11%; Not being home: 9%; Abandonment: 6%; Jealousy: 6%; Capgras: 4%; Erotomania: 3%; Phantom Boarder: 1% Hallucinations: Visual: 4.2%; Auditory+Visual: 2%; Auditory: 1%; Tactile: 0.4%; Olfactory: 0.1%
Nagahama et al.^11^	DLB	100	Delusions: 25% Hallucinations: 78% Misidentifications: 56%	Delusions: Theft: 14%; Persecutory: 11%; Hypochondriac: 3%; Jealousy: 1%; Pregnancy: 1% Hallucinations: Auditory: 8%; Tactile: 2% Misidentifications: People: 17%; Objects: 14%; Places: 28%; Phantom Boarder: 11%; Reduplication of people: 10%; Reduplication of places: 6%; Capgras: 6%; Television: 3%
Nagahama et al.^39^	AD/DLB	AD: 200 DLB: 200	–	Persecutory delusions: AD: 55%; DLB: 65.5% Visual hallucinations: AD: 0%; DLB: 86.5% Auditory hallucinations: AD: 8%; DLB: 87.5% MI of people AD: 11.5%; DLB: 25% Television sign: AD: 1.5%; DLB: 4% Mirror sign: AD: 3%; DLB: 4.5%
Nedelec-Ciceri et al.^9^	AD	98	Misidentifications: 81.6%	Misidentifications: MI of people: 62% (Capgras: 1%) MI of places: 81% MI of self-identification: 14% (mirror 10%)
Naasan et al.^40^	AD DLB FTD	AD: 111 DLB: 59 FTD: 201	Delusions: AD: 16.2% DLB: 25.4% FTD: 18.4% Hallucinations: AD: 12.6% DLB: 47.5% FTD: 18.1% Psychosis: AD: 22.5% DLB: 56.0% FTD: 20.1%	Delusions: AD – Paranoid: 9.0%; Persecutory: 1.8%; Intrusion: 0.9%; Hurting: 0.9%; Jealousy: 11.1%; Place: 3.0%; Other person: 11.1%; Grandeur: 1.8% DLB – Paranoid: 18.6%; Persecutory: 13.6%; Intrusion: 5.1%; Theft: 3.4%; Hurting: 5.0%; Jealousy: 8.5%; Place: 10.2%; Other person: 14.1%; Grandeur: 8.5% FTD – Paranoid: 11.9%; Persecutory: 7.5%; Intrusion: 2.5%; Theft: 3.1%; Hurting: 3.0%; Jealousy: 7.5%; Place: 1.5%; Other person: 2.5%; Grandeur: 7.5%; Erotomania: 2.5% Hallucinations: AD – Visual: 3.6%; Other hallucinations: 13.5% DLB – Tactile: 3.4%; Olfactory: 1.7%; Visual: 13.5%; Other hallucinations: 47.5% FTD – Tactile: 0.5; Olfactory: 0.5%; Visual: 1.5%; Other hallucinations: 18.1%
Perini et al.^41^	AD/VD/FTD/DLB	186 AD: 146 VD: 13 FTD: 6 DLB: 21	Misidentifications: 33.3-36% AD: 30.1% VD: 46.1% DLB: 57.1% FTD: 0	AD/DLB/VD MI of the house: 28.1/47.6/38.5% Capgras (people): 3.4/33.3/30.8% Capgras (objects/animals): 4.1/9.5/15.4% Fregoli: 0.7/9.5/7.7% Reduplicative Paramnesia: 7.5/23.8/15.4% Intermetamorphosis: 1.4/14.3/15.4% Syndrome of double subjects: 0/4.8%/0 MI of mirror: 4.8%/0/0 Division of people: 8.9/19/15.4% Television: 6.2/19/7.7% Cotard: 0.7%/0/0
Sala et al.^42^	AD	180	Psychosis: 35.5% Delusions: 18.3% Hallucinations: 24% Misidentifications: 11.1%	Delusions: Theft: 11%; Persecution: 6%; Jealousy: 2% Hallucinations: Visual: 21%; Auditory+Visual: 2%; Auditory: 2% Misidentifications: Television: 8%; Phantom Boarder: 2%; Mirror: 1%
Suárez-González et al.^43^	AD/DLB	165 AD: 85 DLB: 80	Delusions AD: 32.9%/DLB: 47.5% Hallucinations AD: 12.9%/DLB: 51.2% Misidentifications: AD: 17.6%/DLB: 42.5%	Delusions (AD/DLB): Paranoid: 20/23.7% Abandonment: 2.3/6.2% Somatic: 0/5% Hallucinations (AD/DLB): Visual: 12.9/50%; Auditory: 1.2/17.5%; Tactile: 0/3.7%; Olfactory: 0/3.7% Misidentifications (AD/DLB): Capgras: 9.4/22.5%; Phantom Boarder: 4.7/22.5%; Not being home: 7.1/31.2%; Television: 3.5/13.7%; Mirror: 1.2/2.5%
Tsunomoda et al.^44^	DLB	124	Delusions: 54.8% Hallucinations: 63.7% Misidentifications: 13.7%	Delusions: Theft: 25.8%; Persecutory: 14.5%; Jealousy: 6.5%; Abandonment: 4% Hallucinations: Visual: 60.5%; Auditory: 35.5%; Auditory+Visual: 32.3% Misidentifications: Phantom Boarder: 31.4%; Not being home: 13.7%; Television: 12.1%; Capgras: 8.9%
Tzeng et al.^45^	DLB	207	Delusions: 51.2%	Delusions: Theft: 35.3%; Persecutory: 21.3%; Not being home: 10.8%; Jealousy: 7.2%; Abandonment: 4.8% Misidentifications: Phantom Boarder: 2.9%; Media people in the house: 2.9%; Capgras: 1%
Wilson et al.^5^	AD	478	Delusions: 27.3% Hallucinations: 29.6% Misidentifications: 25.5%	Hallucinations: Auditory+Visual: 14%; Visual: 11%; Auditory: 5%

AD: Alzheimer’s dementia; VD: vascular dementia; DLB: dementia with Lewy bodies; FTD: frontotemporal dementia; BV: behavioral variant; ALS: amyotrophic lateral sclerosis; PPA: primary progressive aphasia; SD: semantic dementia; CBD: corticobasal degeneration; PSP: progressive supranuclear palsy; PD: Parkinson’s disease; MIs: misidentifications.

Regarding the place of origin of the sample, 21 (72.5%) studies were carried out with community-dwelling older adults, 3 (10%) were conducted with hospitalized patients, 2 (7%) were developed in several environments, 1 (3.5%) in a long-permanence institution for older adults, and 2 (7%) did not mention the origin of the patients (autopsy). In general, the female gender was more prevalent in the studies.

In relation to the assessment of the psychotic symptoms, clinical interview and/or review of medical charts were the most commonly used, in 10 (35%) studies. The others used the following: the Neuropsychiatric Inventory (NPI) in 9 (31%) studies, the *DSM* criteria (10%; n=3), the Behavioral Pathology in Alzheimer’s Disease (BEHAVE-AD) scale in 3 (10%) studies, several scales (7%; n=2), the Cambridge Examination for Mental Disorders of the Elderly (CAMDEX) in 1 (3.5%) study, and the Dementia Psychosis Scale, also in 1 (3.5%) study.

Frequency of delusions was reported in 23 (82%) studies, ranging from 2 to 68.4%. Hallucinations were reported in 17 (61%) studies, with a frequency of 3–32%; and MIs, in 15 (54%) studies, ranging from 5.2 to 81.6%.

Persecutory delusions were reported in 23 (79%) studies, with a frequency of 2–55%. Delusions of jealousy were reported in 19 (66%) studies, with a frequency ranging from 2 to 26%. Delusions of theft were reported in 17 (59%) studies, with a frequency ranging from 2 to 75.5%. Delusions of abandonment were described in 13 (45%) studies, with a frequency of 0.5–37%. Other delusion contents mentioned were the following: grandiose, somatic, poisoning, nihilistic, erotic, and hypochondriac.

The hallucinations most frequently associated with AD were those of the visual and auditory types. Tactile and olfactory hallucinations were also reported, although in lower frequency. Auditory hallucinations were reported in 16 (55%) studies and visual hallucinations in 15 (52%) studies, with frequency values ranging from 1 to 13.3% and 3 to 25%, respectively. Tactile hallucinations were reported in 6 (21%) studies with a frequency of 0.2–1%, olfactory hallucinations in 1 (3.5%) study with a frequency of 0.1%, and gustatory hallucinations in 1 (3.5%) study with a frequency of 0.5%.

Regarding MIs, imposter delusion was the most frequently documented in 17 (59%) articles, with a frequency of 0.6–37%. It was followed by phantom boarder, mentioned in 13 (45%) articles, with a frequency of 1–21%. The “not being home” delusion was mentioned in 11 (38%) articles, with a frequency of 3.1–47%. Mirrored-self MI was mentioned in 9 (31%) articles and television delusion was mentioned in 8 (28%) articles, both with a frequency of 0.5–8%. Other MIs mentioned were those of media people in the house, reduplication of people and places, and Cotard’s syndrome (walking corpse syndrome).

Associations of the psychotic symptoms with more severe dementia conditions were cited, with more mortality and aggressive behaviors^23^. Two studies referenced the relationship between psychotic symptoms and anatomical changes, finding the relationship between delusions and left temporal atrophy. Auditory hallucinations were associated with a greater number of neurons in the hippocampal gyrus and a smaller number of cells in the dorsal raphe nucleus and parahippocampal gyrus^24,25^.

### Dementia with Lewy bodies

Nine articles on DLB were included (Table 1): 7 (78%) were carried out with community-dwelling patients, 1 (11%) with hospitalized patients, and 1 (11%) did not indicate the place (autopsy). The samples presented similar mean age values and predominance of the female gender. The criteria for the diagnosis of DLB varied according to the studies, with the following being cited: *Dementia with Lewy Bodies Consortium* (n=4; 45%), clinical evaluation (n=3; 33%), *DSM-IV* (n=1; 11%), and autopsy (n=1; 11%).

Regarding the psychotic symptoms, 6 (67%) studies used the NPI, 2 (22%) studies used a semi-structured interview, and 1 (11%) resorted to a medical record review to assess them.

Delusions in general were reported with frequency values ranging from 9.5 to 54.8% in 6 (57%) studies. Persecutory delusions were reported in 5 (71%) studies, ranging from 11 to 65.5%. Delusions of theft were reported in 3 (43%) studies, with a frequency ranging from 14 to 35.3%. Delusions of jealousy were reported in 3 (43%) studies, with a frequency of 1–7.2%. Delusion of abandonment was reported in 3 (43%) studies, with a frequency of 4–6.2%. Other delusions reported were hypochondriac and pregnancy-related.

Hallucinations in total were reported in 5 (56%) studies, with a frequency of 11 and 79.5%, with higher frequency values being of the visual and auditory types. Auditory hallucinations were reported in four studies (from 8 to 87.5%) and visual hallucinations in four studies (from 13.5 to 86.5%). Tactile hallucinations were also reported in four studies with frequency values from 2 to 4.1%, and olfactory hallucinations were present in three studies with frequencies between 1.7 and 7.3%.

MIs were addressed in a general manner in 5 (71%) studies, with frequency values from 13.7 to 57.1%. Imposter syndrome was documented in 5 (71%) studies (from 1 to 33.3%). The idea of recognizing close people on television was present in 5 (71%) studies (from 3 to 19%). Phantom boarder was present in 4 (57%) studies, with frequency values between 3 and 31.4%. The idea of not being home was present in 3 (43%) studies with a frequency of 10.8–31.2%, and the mirror sign was present in 2 (29%) studies with a frequency of 2.5–4.5%. Other MIs cited were reduplication of people and places, in addition to the idea of media people in the house.

Female gender was associated with hallucinations in one study and with delusions in another^44^. One of the studies reported the relationship between delusions and the severity of the disease, caregiver burden, the presence of visual hallucinations, and more irritability and agitation^45^.

### Vascular dementia

Five studies addressed VD (Table 1), with 4 (80%) of them conducted in the community and 1 (20%) in a hospital setting. There was a predominance of the male gender in the samples. In addition, 3 (60%) studies used the *DSM* criteria for diagnosis, and 2 (40%) resorted to clinical evaluations. Regarding the assessment of the psychotic symptoms, 2 (40%) studies used the NPI, 2 (40%) resorted to clinical interviews, and 1 (20%) employed the BEHAVE-AD scale.

When considering frequency in general, delusions and MIs were mentioned in 3 (60%) studies, and hallucinations were mentioned in 2 (40%) studies. The frequency values were as follows: 12.5–50% for delusions, 6.8–46% for MIs, and 15.5–27% for hallucinations. The most cited subtypes of delusions and their respective frequency values were as follows: delusion of theft in three studies (from 6.8 to 23%), delusion of abandonment in two studies (from 3.4 to 7%), delusion of jealousy in two studies (from 3 to 14%), persecutory delusion in one study (23%), and somatic delusion in one study (3%).

In relation to the hallucinations, only those of the auditory and visual types were cited in two studies, with frequency values ranging from 7 to 20%. MIs were cited in three studies and were the following: not being home (from 3 to 39%), imposter syndrome (from 5 to 30%), and phantom boarder (from 5 to 14%). Two studies cited the television signal (from 2 to 8%).

### Frontotemporal dementia

Six articles addressed FTD (Table 1). Most of the studies were conducted in the community with samples ranging from 6 to 88 subjects. There was a predominance of the male gender in the samples. The diagnosis of FTD was through clinical evaluation in half of the studies, and through autopsy and international consensus in the others.

The psychotic symptoms were assessed through the NPI in half of the studies and by means of clinical evaluations in the other half, with a similar frequency of delusions and hallucinations in two studies, approximately 3–25%. The presence of persecutory delusions (from 1 to 18%), somatic delusions (9%), delusions of grandeur (7.5%), and delusions of jealousy (7.5%) were cited. A combination of the delusions was reported in 38% of the patients. The hallucinations mentioned were as follows: auditory, with a frequency of 9%; visual, with 13.3%; and tactile, with a frequency of 4%. Four studies highlighted the absence of a relationship between MI and FTD.

Psychotic symptoms were associated with volume loss in cortical and subcortical networks, as well as with the presence of the *C9orf72* gene.

The percentage of studies reporting psychotic symptoms and the variation in frequency of psychotic symptom subtypes in the different types of dementia is presented in Table 2.

**Table 2.** Frequency of studies reporting psychotic symptoms and types of symptoms according to the etiology of dementia.

Type of dementia	Psychotic symptoms (%)	Delusions (%)	Hallucinations (%)	Misidentifications (%)
Alzheimer’s disease	82	2–68.4	3–32	5.2–81.6
Dementia with Lewy bodies	71	9.5–54.8	11–79.5	13.7–57.1
Vascular dementia	60	12.5–50	15.5–27	6.8–46
Frontotemporal dementia	66	3–25	3–25	0

## DISCUSSION

This systematic review identified a frequency of 34–63% of psychotic symptoms in general in dementia conditions of the most diverse etiologies; these numbers are corroborated by the literature, since approximately 60% of the patients with dementia suffer from one or more behavioral changes^32^.

The articles in this review met at least 70% of the STROBE items. Most of the studies detailed the criteria used for diagnosing dementia and the behavioral changes and provided details of the methods, which assisted in better understanding the results. It is important to point out that none of the studies included performed a sample calculation and that a vast majority did not detail sample selection through a flowchart.

In AD, delusions were the most commonly reported psychotic symptoms in different studies, especially those of persecution, jealousy, theft, and abandonment. Some delusions presented a wide variation in frequency, such as the delusion of theft, ranging from 2^24^ to 75.5%^29^. A possible explanation for this difference can be the methods used to assess the symptoms. Förstl et al.^24^ used the CAMDEX scale, while Ikeda et al.^29^ employed a structured interview with the caregiver based on a delusions subscale of the NPI. The delusions of jealousy varied from 2 to 26%. Delusions presented in around 35% people with AD, and infidelity is one of the most common types^46^.

The difference observed in the frequency of the abandonment delusion can also be explained by the method used to assess the psychotic symptoms. The highest and lowest frequency values were found in a study that applied several scales and in another that only conducted a review of medical records, respectively^7,34–37^.

In relation to AD, hallucinations were reported in more than half of the studies, with a predominance of the visual and auditory types in terms of frequency. Regarding the MIs, they were mentioned in at least half of the studies, mainly the imposter syndrome, “not being home,” phantom boarder, TV delusion, and mirrored-self MI. The most frequently described instrument in the assessment of the psychotic symptoms in AD was the NPI in 31% of the studies, but clinical assessment was the most used method. The NPI is a semi-structured interview carried out with the caregiver to investigate delusions and hallucinations in addition to other behavioral symptoms^47^; therefore, the absence of a real description of the psychotic symptoms due to an inadequate approach can be considered a bias in the studies.

In DLB, delusions were reported in up to 78% of the studies included, with the following types being more described: persecution, theft, jealousy, and abandonment, that is, the same content as the delusions found in AD. This finding is in line with other studies that report delusions as the main neuropsychiatric symptoms in patients with DLB^45^. Hallucinations in DLB were reported in six of the studies, with the visual and auditory types having the highest frequency, and visual hallucinations are part of the diagnostic criteria for this type of dementia^8^. This could be a bias as hallucinations in other modalities are part of the clinic of this type of dementia^8^. MIs were also found in seven of the studies on DLB, with predominance of description of the following types: imposter syndrome, recognizing people on television, phantom boarder, ideas of not being at home, and mirrored-self MI. MIs appeared in a greater proportion of studies on DLB than on AD, which is in agreement with the literature^48^.

Regarding VD, delusions and MIs were mentioned in 60% of the studies, and hallucinations were mentioned in 40%. The most commonly mentioned delusion contents were those of the theft, abandonment, and jealousy type. The most common MIs were “not being home,” imposter syndrome, and phantom boarder. The only hallucinations reported were those of the auditory and visual types. Our findings were in agreement with the literature regarding the fact that there are no differences between psychosis in AD and VD^49^.

FTD was addressed in six studies. Persecutory, somatic, grandeur, and jealousy delusions were reported. The hallucinations cited were of the auditory and visual types, although with prevalence values below 14%. MIs were not reported in all the studies. This can be explained by the variable accuracy in the clinical diagnosis of FTD, in addition to the difficulty differentiating between psychotic symptoms and behavioral changes characteristic of the condition^6^. According to the data found, it would be important to pay more attention to other findings of the disease in the diagnosis of FTD, such as apathy, disinhibition, and aberrant motor behavior, and less to psychotic symptoms^50^.

In general, AD presents more delusions than hallucinations. On the contrary, DLB triggers more hallucinations, even auditory ones, when compared to other types of dementia and also presents a concomitantly high frequency of delusions. VD presents fewer psychotic symptoms than DLB and AD, but this may be a bias due to the greater number of studies on these types of dementia. Regarding the content of the delusions, it was not possible to differentiate the etiology of dementia between AD, DLB, or VD, with greater presence of persecutory delusions. FTD was the only etiology not associated with delusions of abandonment and associated with the presence of delusions of grandeur^21^, despite the low frequency when compared to the other causes. Tactile and olfactory hallucinations are not common in any of the dementias. The presence of MI does not allow for differentiating the dementias. It is also important to emphasize the absence of MIs in cases of FTD^28,41^. Despite the high association between psychotic symptoms and dementia, there is still a gap in the differential diagnosis between primary and cognition-related conditions^51^. Approximately 60% of patients with late-onset psychotic symptoms have secondary psychosis, and a thorough medical evaluation is extremely important^52^.

Unfortunately, no studies were found with a neuropsychiatric symptom’s description of other causes of dementia, such as secondary to the use of alcohol, due to traumatic brain injury, and others. It is suggested to conduct research studies on this theme not only in the most known neurocognitive disorders but also in conditions with other etiologies that are not less important.

Another limitation would be the use of different definitions of dementia and of the individual psychotic phenomena^32^. This difficulty was found in our review, as some studies did not separate, for example, MI symptoms from delusional symptoms. The absence of well-defined criteria for the concept of delusion in patients with neurocognitive disorder can lead to this wide variation in the reported frequency of these symptoms^53^. Another consideration is the lack of insight and memory impairment of the patients with dementia, which can hinder data collection^16^. It is also necessary to pay attention to the description of the reported delusion, as in the case of the imposter delusion, which presented a lower frequency when it was evaluated exclusively with respect to the caregiver^7^. The term neuropsychiatric symptoms was not used in the search, so it may be that we did not include articles that reported psychotic symptoms. In addition, many instruments used for neurocognitive conditions in clinical practice do not include questions about psychosis^51^.

The population studied has both cognitive and psychotic symptoms, clinical features that are underdiagnosed. For the diagnosis of psychotic symptoms, the clinical interview and the review of medical records were used in a large percentage of the studies included. Al-Huthail^54^ reported an accuracy of 0% for psychotic symptoms when comparing the diagnosis of physicians of others specialties with psychiatrists. The difficulty in documenting psychosis also increases when considering that patients and family members may not report these symptoms for fear of being stigmatized^51^. Many of the scales used were filled with information provided by caregivers, generating some interference, such as caregiver overload, personality, and the ability to perceive changes in the patient’s behavior^55^.

Appropriate classification of the psychotic symptoms is important to clarify the biological mechanisms and to develop new therapies for such symptoms, especially in dementia^47^. However, there is still a gap in the literature on the types of symptoms of the BPSD. However, the strongest point of this study is its originality, since there are no systematic reviews in the literature addressing the psychotic symptoms in dementia, both in specific cases and in different etiologies.

The psychotic symptoms presented high frequency in dementia conditions of the most varied etiologies, with the exception of FTD. The high prevalence demonstrates the importance of clinical investigation and the use of scales that address psychotic symptoms in patients with neurocognitive conditions, regardless of etiology or severity. Future studies about the neuropsychiatric symptoms of dementia conditions are suggested, mainly not of so classic etiologies, such as the alcoholic one. Moreover, the frequency of psychotic symptoms in dementia varied widely among studies, and a meta-analysis could help in identifying its prevalence.
